# Neurocognitive Changes among Elderly Exposed to PCBs/PCDFs in Taiwan

**DOI:** 10.1289/ehp.10134

**Published:** 2007-10-16

**Authors:** Kao-Chang Lin, Nai-Wen Guo, Pei-Chien Tsai, Chiu-Yueh Yang, YueLiang Leon Guo

**Affiliations:** 1 Department of Neurology, Chi-Mei Medical Center, Tainan, Taiwan; 2 Institute of Behavioral Medicine and; 3 Institute of Basic Medical Sciences, National Chen-Kung University Medical College, Tainan, Taiwan; 4 Department of Environmental and Occupational Medicine, National Taiwan University College of Medicine, Taipei, Taiwan; 5 Department of Environmental and Occupational Medicine, National Taiwan University Hospital, Taipei, Taiwan

**Keywords:** elderly, neurocognitive functioning, neuropsychological tests, PCBs, PCDFs, polychlorinated biphenyls, polychlorinated dibenzofurans

## Abstract

**Background:**

In 1979 approximately 2,000 people were exposed to polychlorinated biphenyls (PCBs) and polychlorinated dibenzofurans (PCDFs) due to ingestion of contaminated cooking oil in Taiwan. Although a previous study has shown delayed developmental milestones and poorer neurocognitive functioning in children born to exposed mothers, it is unclear whether neurocognitive functioning was impaired in people who were directly exposed to the PCBs and PDCFs.

**Objective:**

The objective of this study was to compare neurocognitive functioning in people exposed to PCBs and PCDFs with that of unexposed sex- and age-matched neighbors.

**Methods:**

We conducted a retrospective cohort study among exposed and unexposed subjects ≥60 years of age using prospective outcome measurements. We evaluated neurocognitive tests including cognition, memory modalities, learning, motor and sensory function, mood, and daily activity.

**Results:**

In total, 162 (59%) exposed and 151 (55%) reference subjects completed this study. In exposed men, all test results were similar to the reference group; however, exposed women had reduced functioning in attention and digit span (ADS), visual memory span (VMS), and verbal memory recalls (VMR), especially learning ability. We also found a borderline reduction in the Mini-Mental State Examination. The digit symbol, motor, sensory, depression (determined by the Geriatric Depression Scale-Short Form), and activity of daily life were not different between the exposed and reference groups. A significant dose–response relationship was found for VMR, ADS, and VMS.

**Conclusion:**

Our study showed dose-dependent neurocognitive deficits in certain aspects of attention, visual memory, and learning ability in women previously exposed to PCBs and PCDFs, but not in exposed men.

Polychlorinated biphenyls (PCBs) are toxic chemicals that have been widely used throughout the world [Agency for Toxic Substances and Disease Registry (ATSDR) 1997; Babich 1998]. PCB congeners have been reported to cause variable toxicity in animals. Major human events of heavy exposure occurred in Japan in 1968 (known as “Yusho”) and in Taiwan in 1979 (known as “Yucheng”), both as results of ingestion of contaminated rice oil (Hsu et al. 1985; Masuda et al. 1982). In both events, heat-degradation products of PCBs—polychlorinated dibenzofurans (PCDFs)—were found to be responsible for the toxicities associated with the exposure (Guo et al. 1997; Lambert et al. 2006; Masuda et al. 1982). In Taiwan, people who consumed the contaminated rice oil suffered from general malaise, chloracne, peripheral neuropathy, and headache (Chen et al. 1981). Serial follow-up of the exposed people disclosed further adverse health consequences, such as thyroid goiter, menstruation and reproductive problems (Guo YL et al. 1999; Yu et al. 2000), and several health and cognitive outcomes in their descendents (Guo et al. 2004).

In rats, PCBs have been reported to have a profound effect on the nervous system. Seegal (1996) reported that one mixture of PCBs (Aroclor 1260) lowered dopamine concentration and interfered with neurotransmitters, and in turn affected animal behavior, and induced slow kinetic movements. Schantz and Widholm (2001) found that adult rats fed mixed PCBs or Aroclor 1260 had impaired learning ability and behavioral withdrawal from stimuli. Animals fed lower-chlorinated PCBs have been found to have spatial discrimination impairment and cubic conceptual disruption (Mele et al. 1986; Schantz et al. 1989; Seegal et al. 1991).

PCBs have also been shown to exert neurobehavioral effects on the second generation, as well as in those who were directly exposed. In a study by Bowman and Heironimus (1981), pregnant rhesus monkeys fed Aroclor 1248 delivered baby monkeys that had impaired learning ability and behavior at 6-, 12-, and 44-month follow-up. In humans, children born to exposed mothers in Yucheng had lower scores by several different measurements for neurocognitive functioning than did children of unexposed mothers (Chen et al. 1992; Guo et al. 1995a, 1995c). Jacobson et al. (1996) reported that the intelligence quotient at 11 years of age was lower in children born to mothers who consumed Lake Michigan sport fish and who had elevated serum PCBs. Also, in the follow-up of an adult cohort of Lake Michigan fish eaters, exposure to PCBs was associated with impaired neuropsychological tests in certain areas of memory and learning (Schantz 1996; Schantz et al. 2001). However, it is unknown whether neurocognitive function is affected in members of the Yucheng population exposed to PCBs and PCDFs as adults. Therefore, we conducted an evaluation to determine whether neurocognitive function was impaired in the PCB/PCDF-exposed elderly.

## Methods

### Subjects

**Figure 1:**
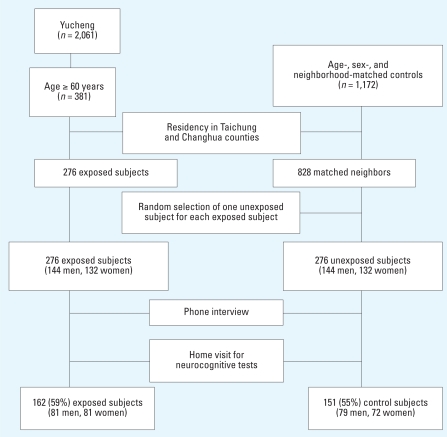
Enrollment of the PCBs study subjects and reference group. Only those residing in the selected 10 townships of Taichung and Changhua counties were included as candidates.

**Figure 2:**
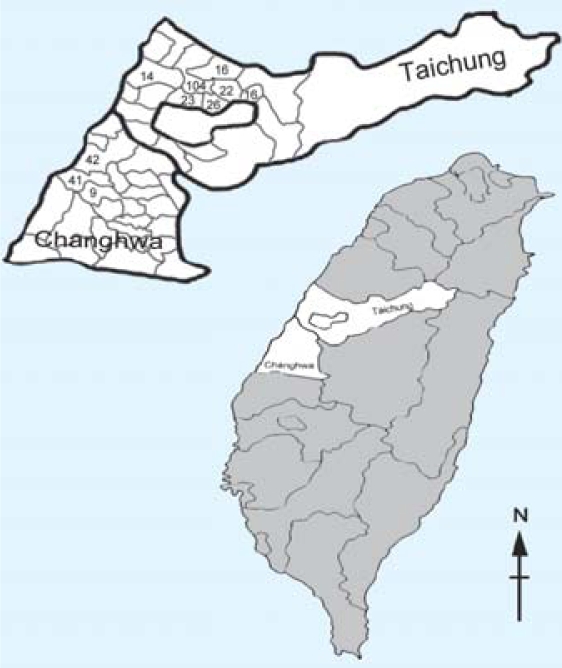
The distribution of the study population in Taichung and Changhua counties in central Taiwan. Numbers indicate the number of study subjects per township.

We conducted a cohort study with prospective outcome measurements among members of an existing cohort from the 1979 exposure in midcentral Taiwan (the Yucheng cohort), using their sex- and age-matched neighbors as a reference group. This study was approved by the Committee for Human Research at Cheng-Kung University Medical College, and all subjects provided written informed consent. The details of the Yucheng cohort and the matched unexposed subjects have been detailed previously (Guo YL et al. 1999). Exposure had ended for all subjects in 1980 with notification of the Yucheng event. For the neurocognitive functioning study, we recruited only Yucheng and unexposed subjects ≥60 years of age by 1 July 2002. Figure 1 shows the recruitment and participation of study subjects. Among the 381 Yucheng subjects ≥60 years of age, 276 were alive and resided in 10 townships in central Taiwan (Figure 2); we selected these 276 subjects as candidates for this study. For each Yucheng subject, 3 sex- and age-matched (within 3 years) unexposed individuals had been previously identified from the same neighborhoods (Guo YL et al. 1999). Among these 3 potential reference subjects, only 1 was randomly selected as a comparison subject. Beginning July 2002, a structured questionnaire was administered by phone interview (taking approximately 30 min) and included demographics, habits, medical history, and general health status. At the end of interview, subjects were invited to participate in a neurocognitive examination by home visit, and oral informed consent was obtained. Among those who agreed to participate, neurocognitive tests were administered by interviewers during home visits. Only those who completed both phone interview and neurocognitive examination were included in the final analysis. The study ended in 2004.

In 1980–1982, serum PCB levels were analyzed in approximately 80% of Yucheng individuals using packed column, electron-capture gas chromatography and the Webb–McCall method adapted to a computerized data system; a Japanese PCB mixture (Kanechlor 500) was used as a reference standard (Tsai et al. 2006).

### Neurocognitive testing

For neurocognitive evaluation, we used a standardized neurobehavioral battery consisting of 10 tests, including intelligence, verbal, learning and memory, visual-constructive and organizational skills, sensory tactile function, and motor performance (Golden 1987; Lezak 1995). All tests were in Chinese and have been used previously in Taiwan with good reproducibility and validity (Guo NW et al. 1999; Yu et al. 1998). We used the Mini-Mental State Examination (MMSE), with a possible total score of 30, to assess global cortical function, including orientation, attention, immediate and short-term recall, language, and the ability to follow simple verbal and written commands (Francesco et al. 1999). Attention and digit span (ADS) of the Wechsler Adult Intelligence Scale (WAIS) was used to measure immediate learning memory. The ADS comprises two subtests, forward and backward, which involve different cognitive processes, and is similar to the trail-making test for attention and concentration (Fiedler et al. 1996). The digit symbol (DS) subset was used to assess mental flexibility, executive functioning, and visual scanning. The DS contains a list of numbers that are associated with certain symbols and a list of random digits from 1 through 7, with blank squares below each digit; the score is the total number of correct symbols completed within 2 min (Wechsler 1981).

We used five trials of the verbal memory recall (VMR) to assess learning and short-term memory. Each trial was composed of 10 items of different categories. Thirty minutes after finishing five trials, a delayed recall test was given without warning. Learning capacity was calculated by subtracting the score of the first trial from that of the last trial, with the total amount of the differences defined as learning ability. The purpose of and the capacity tested by the VMR test were similar to those of the California Verbal Learning Test (CVLT; Delise 1987) by Schantz et al. (2001). We used the Visual Memory Span (VMS) from the Wechsler Memory Scale-Revised to test for recall; a visual display of eight boards (red and green cards) were tapped forward and backward in sequences, and recall was scored (total potential score of 24) (Schenck 2000). We used a Finger-Tapping serial (10 trials, with hands alternating from one to the other), which is similar to a pegboard test, to measure the timing of motor performance. Sensory Tactile Performance was used to assess sensorial perceptive function based on modified Luria’s criteria (Golden 1987); the assessment included the following tests: light touch, pin-prick, proprioception, two-point discrimination, and graphic-writing sensation on small and large fiber tactile. We used the Geriatric Depression Scale-Short Form (GDS-S) as a measure of depressive symptomatology (Alden et al. 1989). The Activity of Daily Life (ADL) scale was used to understand normal motor activity and daily living conditions (Van der Putten et al. 1999). All of the above-selected procedures have been previously tested for validity (Hartman 1995). We estimated that the testing battery could be completed in 90 min.

We invited registered nurses who were receiving continuing education in central Taiwan to administer the neuropsychological tests for this study, and 20 responded. The nurses were trained by a clinical psychologist (N.W.G.) and a neurologist (K.C.L.), and training included 12 hr of lectures followed by 8 hr of practice. Before the pilot study, the potential interviewers took a written test. Pilot test sessions were carried out on 20 subjects not included in the study. Ten trainees failed to fulfill the required standards and thus were not included as interviewers in this study. For the home visit and neurocognitive tests, each interviewer was assigned matched subjects (one Yucheng subject and one reference subject) on the same day of testing. The interviewers were blinded to the subjects’ exposure status.

### Statistical analysis

All test results were reviewed for possible poor performance or recording error before data processing by a clinical psychologist (N.W.G.). Subjects who did not complete all testing items were excluded from the analysis. Therefore, data for 162 exposed subjects (59%) and 151 reference subjects (55%) were analyzed. Although unexposed neighbors were matched to the exposed individuals by age and sex, the analysis was performed in an unmatched manner (Table 1).

A total score was calculated for each neurocognitive test (including the MMSE, DS, ADS, Sensory Tactile Performance, VMS, Finger-Tapping serial, and GDS-S). A higher score indicated a better result for all tests, except for the Finger-Tapping serial and GDS-S. We used the Student’s *t*-test and chi-square test to determine differences in the demographics between exposed and reference groups. Multiple regression analyses were used to adjust for age and education. The adjusted values were then compared between exposed and reference groups. Because of a large difference of neurocognitive effects due to exposure across sexes, the analyses were performed separately for men and women. Yucheng individuals were grouped into high-, medium-, and low-exposure groups according to serum PCB levels measured in 1980–1982. We used a test for linear trend to examine dose response by coding unexposed as 0, those with 1980–1982 serum PCB levels of < 35.0 ppb as 1, those with PCB levels of 35.0–95.9 ppb as 2, and those with PCB levels ≥96.0 ppb as 3. A linear regression was performed using age- and education-adjusted neurocognitive scores as dependent variables and the exposure group as an independent variable. All tests for significance were two-sided.

## Results

The field work for this study was conducted from July 2002 to January 2004. Figure 1 shows the process of recruitment and testing of subjects. A total of 313 eligible participants were enrolled in the study, 59% of the candidates in the exposed group and 55% in the reference group. The average age (± SD) was 69.5 ± 5.9 years (range, 60.0–91.1 years), and the educational level was 4.3 ± 3.5 years (primary school is 6 years, and secondary is 3 years). The reference group was of similar age and education level. Height, body weight, body mass index (BMI), and self-reported smoking and alcohol use were not statistically different between exposed and reference groups (Table 2).

The average time for neurocognitive testing was 72.9 ± 22.1 min; this was similar between exposed and reference groups. Test–retest was performed 4 months after the first test in a random sampling of 20 participants, with reliability of 0.85 for MMSE, 0.66 for DS, 0.69 for ADS, 0.73 for VMS, and 0.77 for VMS delayed recall. Most of the tests were inversely related to age and directly related to education; thus scores were adjusted for age and years of education. Smoking, alcohol use, BMI, and other covariates were not related to the test scores. In men, exposure to PCBs/PCDFs did not significantly affect any of the testing scores. In contrast, exposed women scored lower than the reference group in MMSE, VMR, ADS, and VMS, but their scores were similar to those of the reference group in DS, finger tapping, sensory scores, depression scores, and ADL (Table 3). We also found a borderline significant decrease in MMSE in Yucheng women.

We examined the dose–response relationship by dividing the Yucheng women into low- (< 35 ppb), intermediate- (35–95 ppb), and high-exposure (> 95 ppb) groups according to the 1980–1982 PCB levels. We found significant linear trends in the reduction in VMR, ADS, and VMS when reference, low-, intermediate-, and high-exposure groups were compared (Table 4).

## Discussion

The present study provides evidence that previous exposure to PCBs and PCDFs in adulthood caused neurocognitive declines, particularly in learning and memory, 25 years after exposure. Such damage was found in women who were, on average, in their late sixties. Despite the limited sample size, we found reduced capability in memory and learning in a dose-dependent manner in Yucheng women.

The damage to the central nervous system observed in this study leads to our postulation that exposure to PCBs and PCDFs had an influence on the vulnerable deep-seated hippocampus but not on the the durable executive or sensory parts of the cortical brain. Although different tasks are involved, the ADS, VMR, and VMS all require immediate and short-term working memory, which appeared to be affected by PCBs/PCDFs in the Yucheng women. Our data show a relevant significance of working memory deficit rather than executive motor and sensorial performance.

To date, there has been only one publication about cognitive function in PCB-exposed elderly (Schantz et al. 2001).The study included a medium-sized sample (exposed, 101; referent, 78) and found three positive results in cognitive deficits, with linear dose response. However, sex-specific effects were not examined. The cohort of sport-caught–fish eaters had a mean calculated PCB level of 7.9 ng/g cumulative dose in the blood (Table 5). In the present study, we included more subjects (exposed, 162; referent, 151), with higher initial outbreak exposure [mean of 78.3 ppb (ng/g)], sex-specific neurocognitive effects, and a significant dose response in the ADS, VMS, and VMR in the exposed females.

Age and education are well known to affect neurocognitive functioning (Anger 2000; Leckliter and Matarrazo 1989; Zhou et al. 2002). In the present study, most tests were inversely associated with increasing age and were directly associated with longer years of education in both men and women. Yucheng women had lower performance in the ADS, VMR, and VMS in crude analyses, as well as analyses adjusted for and age and education.

Complete neuropsychological tests, such as the Wechsler Adult Intelligence Scale-Revised (WAIS-R; Wechsler 1981), the Halstead-Reitan Battery (Leckliter and Matarrazo 1989), and Luria-Nebraska’s tests (Golden 1987), are difficult to complete in epidemiologic studies. We selected a subset of test items, a common practice in many investigations. In addition to screening neuropsychological tests for cognition (MMSE), general functioning (ADL), sensory (Luria’s Sensory Scores and 2-point discrimination) and motor (finger tapping serials) ability, we assessed memory function. The MMSE has been used extensively as a screening tool for neurocognitive impairments. The MMSE scores in our reference group were similar to data from people in northern Taiwan but slightly higher than data from southern Taiwan (Liu et al. 1994, 1998). We presume that variable cultural and learning differences may render the MMSE hard to use as a sensitive indicator of a toxic effect.

Depression can potentially affect neurocognitive functioning. We examined the effects of the GDS-S score on neurocognitive test performance and found no significant effects. In addition, Yucheng men and women did not have higher GDS-S scores than the reference group.

Few studies have examined neurocognitive effects of human adult exposure to PCBs and related chemicals. Table 5 shows a comparison of our findings with the results of Schantz et al. (2001). In their study, they compared neurocognitive function between 101 fish eaters and 79 reference subjects. The mean serum level of PCBs was 7.9 ng/g (equivalent to parts per billion used in the present study) among fish eaters several weeks after the neurocognitive evaluation; this was probably severalfold lower than PCB levels in 1980–1982 when our cohort was established. Although no direct comparison has been made between the two exposed groups, the peak serum levels among fish eaters (Schantz et al. 2001) were probably comparable to, or somewhat lower than, the PCB levels in Yucheng subjects. However, fish eaters were exposed to many other contaminants from lake fish, and Yucheng victims were also exposed to PCDFs. In fish eaters, the delayed recall in WMS, semantic-cluster ratio, and List A of trial I in the CVLT were related to PCB exposure, but visual memory was not. Our findings of dose-dependent impairments in VMR among Yucheng women (present study) are comparable to the findings of Schantz et al. (2001). However, we found impaired VMS and ADS in Yucheng women. Whether these impairments were caused by different susceptibility because of genetic background or by the additional effects of PCDFs remains to be determined.

Because we focused on people ≥60 years of age, some of the Yucheng subjects with the highest exposure may have died before the neurocognitive outcomes were assessed, and these subjects may have been most affected with respect to neurocognitive functioning. Therefore, in the present study, we may have underestimated the overall neurocognitive effects that Yucheng exposure could have caused.

In previous studies of the Yucheng cohort, neurocognitive effects were found among children prenatally exposed to PCBs and PCDFs (Chen et al. 1992), likely through trans-placental exposure. Such effects were observed up to the age of 7 years (Guo et al. 1994). However, these findings were not universally compatible with other investigations; for example, Jacobson and Jacobson (1996) found reduced intelligence among children born to mothers who were highly exposed to PCBs, but Gray and co-workers (2005) found intelligence to be unrelated to prenatal PCB exposure. The findings of neurocognitive damage among Yucheng children demonstrate a perplexing contrast to the findings of the present study: Among Yucheng children, boys were more severely affected in their visuospatial capability than girls by Raven’s Progressive Matrices (Guo et al. 1995b). More prominent neurocognitive effects of PCBs/PCDFs on adult women, as well as those on prenatally exposed boys, warrant further investigation from a neurohormonal perspective.

In the present study, we found sex-related damage of memory and learning among Yucheng women. Although the mean serum PCB level in Yucheng women (88.7 ± 106.2 ppb, mean ± SD) was higher than that in Yucheng men (67.4 ± 62.3 ppb), the difference was not statistically significant, and probably did not account for the sex-difference in neurocognitive effects of individuals. This is further evidenced in the Yucheng women exposed to intermediate levels (35–95 ppb); the mean serum level (59.7 ppb) was lower than that in average Yucheng men, but the neurocognitive effects were greater. In a study of randomly selected Swedish people, Herlitz et al. (1997) found that women outperformed men in episodic memory, including verbal memory. Our finding showed higher VMR, learning ability, and delayed recall (Table 3) in reference women than in reference men. However, Yucheng women had decreased VMR, learning, and delayed recall down to the level of the reference men, or even lower, suggesting masculinization of such memory performance among Yucheng women. In contrast, reference men performed better in VMS, which is compatible with the male advantage in visuospatial tasks. Yucheng women had a further drop in VMS. As for ADS, reference men did better than women, but the Yucheng exposure caused a further drop among women. Mechanisms of such sex-related toxic effects remain to be elucidated.

In summary, among humans previously exposed to relatively high levels of PCBs and PCDFs, we identified neurocognitive hazards, especially memory and learning impairments in women.

## Figures and Tables

**Table 1:** Sex, age, education, and PCB level in participants and nonparticipants.

Variable	Participants (*n* = 313)	Nonparticipants (*n* = 239)	*p*-Value
No. participating (% male)			
Exposed	162 (50)	114 (55)	0.39
Control	151 (52)	125 (52)	0.96
Age [years (mean ± SD)]			
Exposed	68.9 ± 5.8	71.4 ± 8.1	< 0.001^a^
Control	69.7 ± 5.4	71.8 ± 8.0	< 0.001^a^
Education [years (mean ± SD)]			
Exposed only	4.0 ± 3.5	3.7 ± 3.1	0.21
PCB level in 1980–1982 [ppb (mean ± SD)]			
Exposed only	78.2 ± 87.6^b^	50.1 ± 55.2^b^	< 0.001^c^

a Student’s *t*-test.

b Data available for 145 participants and 72 nonparticipants.

c Wilcoxon’s rank-sum test.

**Table 2:** Age, education, physical illness, and alcohol and smoking status of Yucheng and reference subjects in Taiwan who took neurocognitive tests.

	Male			Female		
	Yucheng (*n* = 81)	Reference (*n* = 79)	*p*-Value	Yucheng (*n* = 81)	Reference (*n* = 72)	*p*-Value
Age (years)	69.7 ± 6.2	70.0 ± 5.5	0.74	68.1 ± 5.3	69.4 ± 5.3	0.13
Distribution (%)						
60–64	28.4	24.1		34.8	19.4	
65–69	27.2	27.8		28.4	36.1	
70–74	21.0	26.6	0.40	21.0	27.8	0.16
≥ 75	23.4	22.5		14.8	16.7	
Education (years)	5.7 ± 3.9	5.4 ± 2.9	0.65	2.4 ± 3.0	3.3 ± 3.1	0.080
Distribution (%)						
0–3	25.9	22.8		66.7	50.0	
4–6	49.0	65.8		29.6	47.2	
7–9	9.9	6.3	0.08	2.5	2.0	0.10
> 9	14.8	5.1		1.2	0.0	
Current drinking (%)	19.5	27.3	0.29	16.2	21.2	0.48
Current smoking (%)	60.0	52.1	0.37	23.8	30.4	0.39
Body height (cm)	164.3 ± 6.1	163.2 ± 6.1	0.26	155.6 ± 5.7	156.9 ± 4.9	0.15
Body weight (kg)	64.7 ± 8.3	64.2 ± 7.3	0.71	56.7 ± 7.2	56.9 ± 8.0	0.88
BMI (kg/m^2^)	23.8 ± 2.8	24.0 ± 2.4	0.77	23.7 ± 2.9	23.1 ± 2.9	0.28
Serum PCBs in 1980–1982 (ppb, wet base)^a^	67.4 ± 62.3	1.5		88.7 ± 106.2	1.7	

Values shown are mean ± SD or percent. Age, years of education, body height, body weight, and BMI were compared by unpaired *t*-test; age/education distribution, current smoking, and alcohol use were compared by chi-square test.

a Data available for 72 males and 73 females; reference levels were estimated from previous studies in Taiwan.

**Table 3:** Neurocognitive tests of exposed and reference groups, adjusting for age and education.

	Male			Female		
	Yucheng (*n* = 81)	Reference (*n* = 79)	*p*-Value	Yucheng (*n* = 81)	Reference (*n* = 72)	*p*-Value
MMSE	25.6 ± 3.3	25.3 ± 2.7	0.48	21.8 ± 3.9	22.7 ± 4.1	0.069**
VMR	27.7 ± 7.2	27.7 ± 7.9	0.99	26.3 ± 7.4	29.1 ± 7.5	0.024*
Learning ability	3.0 ± 2.7	3.2 ± 3.0	0.71	2.9 ± 3.1	4.1 ± 1.9	0.0052*
30-min delayed recall	5.1 ± 2.7	5.4 ± 2.9	0.55	5.0 ± 2.9	5.8 ± 2.5	0.068*
DS	27.1 ± 11.0	29.2 ± 11.7	0.25	17.7 ± 8.2	19.9 ± 9.8	0.17
ADS	16.8 ± 3.6	17.4 ± 3.7	0.30	13.6 ± 3.4	15.7 ± 3.8	0.0005*
Forward	11.7 ± 2.2	12.3 ± 2.3	0.094	10.5 ± 2.4	11.9 ± 2.3	0.0002*
Backward	5.0 ± 2.2	5.0 ± 2.1	0.99	3.2 ± 1.6	3.8 ± 2.1	0.032*
VMS	12.7 ± 2.6	12.6 ± 2.9	0.96	9.7 ± 2.9	10.8 ± 2.9	0.013*
Forward	7.4 ± 1.5	7.3 ± 1.5	0.83	6.1 ± 1.7	6.6 ± 1.4	0.053**
Backward	5.3 ± 1.5	5.3 ± 1.8	0.92	3.6 ± 1.5	4.2 ± 1.7	0.013*
Finger tapping serials	24.0 ± 7.4	23.1 ± 7.1	0.41	27.9 ± 9.9	26.8 ± 9.7	0.50
Dominant hand	24.3 ± 7.0	24.3 ± 8.6	0.97	29.5 ± 10.3	28.6 ± 11.7	0.59
Nondominant hand	23.8 ± 8.3	21.7 ± 6.0	0.071	26.7 ± 9.9	25.5 ± 9.8	0.47
Luria’s sensory scores						
Dominant hand	53.1 ± 9.1	54.0 ± 10.8	0.58	50.7 ± 7.8	50.2 ± 7.3	0.69
Nondominant hand	42.2 ± 9.0	42.8 ± 8.5	0.71	41.0 ± 7.4	41.7 ± 7.9	0.60
2-point discrimination	13.0 ± 2.3	12.8 ± 2.3	0.62	12.6 ± 2.1	13.0 ± 2.1	0.19
GDS-S	6.7 ± 2.2	6.6 ± 2.0	0.72	6.7 ± 2.1	6.7 ± 1.6	0.82
ADL	96.2 ± 10.3	96.6 ± 9.9	0.78	95.1 ± 9.8	96.7 ± 7.1	0.27

Values shown are mean ± SD.

* < 0.05.

** < 0.1.

**Table 4:** Dose effects of PCB exposure to selected neurocognitive changes in Yucheng women, adjusted for age and years of education.

		Yucheng women by PCB levels in 1980–1982			
	Reference (*n* = 72)	< 35.0 ppb (*n* = 23; mean = 17.2 ppb)	35.0–95.9 ppb (*n* = 27; mean = 59.7 ppb)	≥96.0 ppb (*n* = 23; mean = 194.5 ppb)	*p*-Value for linear trend
VMR	29.1 ± 7.5	26.8 ± 7.3	26.5 ± 6.9	26.1 ± 8.6	0.097**
ADS	15.7 ± 3.8	14.2 ± 4.3	13.3 ± 3.1	13.8 ± 2.9	0.0029*
VMS	10.8 ± 2.9	10.0 ± 2.5	9.7 ± 2.7	9.6 ± 3.2	0.029*

Values shown are mean ± SD.

* < 0.05.

** < 0.1.

**Table 5:** Our results compared with those of Schantz et al. (2001).

	Schantz et al. 2001	This study
Age [range (years)]	64.3 (48–86)	69.5 (60–84)
No. of participants	189	313
Exposed/controls	107/81	162/151
Male/female (%)	42/58	50/50
Route of exposure	Cumulated dose from lake fish	Contaminated cooking oil
PCBs [range (ng/g)]	7.9 (nondetectable–79)	78.3 (3–732)
Other toxicants	Lead, mercury, DDE, dieldrin, etc.	PCDFs
Other toxic effects	No effect	Chloracne, hyperkeratosis, gum pigmentation, etc.
Test results (positive)	Delayed recall, semantic-cluster ratio, list A of trial I in CVLT	VMR (including delayed recalls), ADS, VMS in WAIS-R
Test results (negative)	Pegboard, DS, WCST, HVOT, trail-making A, B	MMSE, DS, ADL sensory, motor performance, GDS-S
Dose-effect	Continuous model using log-transformed serum levels of PCBs and other toxicants	Trend (+) for ADS and visual memory span; borderline (*p* = 0.097) for VMR
Sex-related	Adjusted for sex in most models, but sex-specific effects were not examined	Sex-specific effects were examined; only exposed women were affected

DDE, dichlorodiphenyldichloroethene; HVOT, Hooper Visual Organization Test; WCST, Wisconsin Card Sorting Test.
